# Diagnostic Inflammation Biomarkers for Prediction of 30-Day Mortality Rate in Acute Cholangitis

**DOI:** 10.29337/ijsp.170

**Published:** 2022-03-02

**Authors:** Omer Al-Yahri, Raed M. Al-Zoubi, Azza Alam Elhuda, Amina Ahmad, Mahmood Al Dhaheri, Sherif Abdelaziem, Mustafa Alwani, Ahmad R. Al-Qudimat, Ahmad Zarour

**Affiliations:** 1 Acute care Surgery division, Department of Surgery, Hamad Medical Corporation, Doha, QA; 2 Department of Chemistry, Jordan University of Science and Technology, P.O.Box 3030, Irbid, 22110, Jordan; 3 Surgical Research Section, Department of Surgery, Hamad Medical Corporation, Doha, QA; 4 Department of Biomedical Sciences, QU-Health, College of Health Sciences, Qatar University, Doha, 2713, QA; 5 School of Medicine, Jordan University of Science and Technology, P.O.Box 3030, Irbid, 22110, Jordan, JO

**Keywords:** Acute cholangitis (AC), Mortality, biomarkers, diagnosis, T-bilirubin

## Abstract

**Background:**

Acute cholangitis (AC) is an acute inflammation and infection of the biliary tract, a potentially life-threatening infection, which is usually associated with biliary tree obstruction and impairment of bile flow from the liver to the duodenum. AC is classified by severity from mild, moderate to severe infection (grade I to III, respectively).

**Methods:**

This study recruited a retrospective cohort from Jan 2015 to July 2018. Overall, 294 patients of age ≥ 18 years with AC were enrolled. The study was conducted according to the World Medical Association Declaration of Helsinki. Demographic and laboratory data were collected for analysis. T-Bilirubin and other laboratory results were collected and analyzed using independent *T-test* and ANOVA for continuous values and multivariate COX regression for survival analysis for identifying independent factors for early mortality. The cut-off threshold of T-bilirubin was determined by calculating the area under the receiver operating characteristic (ROC) curve.

**Results:**

There were 213 male and 81 female patients and mean age ± SD of patients was 49.57 ± 16.1 and 56.12 ± 20.18 respectively. 31.9% patients were found older than 60 years of age and 35% patients were found between 30–45 years of age. T-bilirubin and length of hospital stay (LOS) were found statistically significant (P < 0.05) in relation to mortality in AC patients. The area under ROC curve for T-bilirubin level (P = 0.017, OR = 1.010) was 0.717 (95% CI, 6.25–168.9) and this is consistent with the Cut-off point for more than or equal to 38.6 µmol/L (2.26 mg/dL).

**Conclusions:**

In this study, T-bilirubin level is found to be significantly related to short-term mortality in AC. Further studies are still needed with larger cohorts to shed more light on these findings.

**Highlights:**

Herein, we report a retrospective observational study aiming to evaluate biomarkers contributing to mortality in AC and to determine the cut-off diagnostic levels that could be easily used in emergency setting. Overall, 294 patients of age ≥ 18 years with AC were enrolled. The study was conducted according to the World Medical Association Declaration of Helsinki and approved by Institutional Review Board (IRB) with approval: MRC-01-20-823 at Hamad Medical Corporation (HMC). Demographic and laboratory data were collected for analysis. Total-Bilirubin and other laboratory results were collected and analyzed using independent T-test and ANOVA for continuous values and multivariate COX regression for survival analysis for identifying independent factors for early mortality. The cut-off threshold of T-bilirubin was determined by calculating the area under the receiver operating characteristic (ROC) curve. There were 213 male and 81 female patients and mean age ± SD of patients was 49.57 ± 16.1 and 56.12 ± 20.18 respectively. 31.9% patients were found older than 60 years of age and 35% patients were found between 30-45 years of age. T-bilirubin and length of hospital stay (LOS) were found statistically significant (P < 0.05) in relation to mortality in AC patients. The area under ROC curve for T-bilirubin level (P = 0.037, OR = 1.010) was 0.717 (95% CI, 6.25–168.9) and this is consistent with the Cut-off point for more than or equal to 38.6 μmol/L (2.26 mg/dL).

## Introduction

Acute cholangitis (AC), known from the Greek as angiocholitis (angeion:vessels and kholé: bile), is an acute inflammation and infection of the biliary tract, a potentially life-threatening infection that usually associated with biliary tree obstruction and impairment of bile flow from the liver to the duodenum. AC is classified by severity from mild, moderate to severe infection (grade I to III, respectively). Severe acute cholangitis (grade III) is commonly associated with a minimum of one of the neurological, cardiovascular, respiratory, renal, hematological and/or hepatic dysfunctions, that threaten patients’ life without the appropriate management and treatment [1,2].

Late diagnosis and delay in both antimicrobial therapy/biliary decompression have been associated with up to 40% in mortality [3,4]. The management for acute cholangitis is mainly conservative and includes intravenous antibiotics and fluids, cessation of oral feeding. Biliary drainage whether endoscopic retrograde cholangiopancreatography (ERCP) and/or surgical intervention and is vital in many cases specially grade II and III, as the management of AC differs according to its severity grades [5]. Many predictors have been assessed for mortality in AC according to Tokyo guidelines [6] and other related studies [7,8,9] such as age (≥65 year), fever (temperature ≥39°C), disturbance of consciousness, hypertension, serum creatinine (>2 mg/dL), platelet count (<100.000 /mm^3^), hyerbilirubinemia (≥5 mg/dL).

Patients outcomes depend on both severity of AC and time to intervention mentioned previously. The mortality in cholangitis range is between 2.7% and 10% in properly and timely managed AC [10,11,12]. According to 2013/2018 Tokyo guidelines (TG13/TG18), biliary drainage for patients with moderate AC within 48h had 50% reduction in mortality compared to delay in management after 48h [13]. Both white blood cell count (WBC) >10.0 × 10^9^/L and C-reactive protein (CRP) level ≥10 mg/L were used as diagnostic criteria for AC in TG18/13 guidelines [13,14,15]. Finding other useful inflammatory biomarkers for AC is still of great interest for many scholars and commonly markers such as platelet-to-lymphocyte ratio (PLR), neutrophil-to-lymphocyte ratio (NLR), CRP, T-bilirubin, ALP, ALT, Gamma-glutamyl Transferase (GGT), neutrophil and lymphocyte counts have similar discerning powers to WBC. Therefore, more studies to investigate and to evaluate biomarkers in AC patients remain a clinical need. Herein, we report a retrospective observational study aiming to evaluate biomarkers contributing to mortality in AC and to determine the cut-off diagnostic levels that could be easily used in emergency setting.

## Methods

We conducted a retrospective observational study from 2015 to 2018. We included every patient (n = 294) of age ≥ 18 years with AC, who referred to the acute care surgery, Hamad Medical Corporation HMC, Doha, Qatar. Exclusion criteria were any admitted patient of age <18 years. Patients were diagnosed according to Tokyo 2018 guideline criteria [16]. The baseline characteristic data of patient, demographics (gender, age, and BMI), laboratory results (Neutrophil-to-Lymphocyte Ratio (NLR), Platelet-to-lymphocyte Ratio (PLR), white blood cell count (WBC), hemoglobin (HB), neutrophile, lymphocytes, lactic acid, alanine aminotransferase (ALT), aspartate aminotransferase (AST), alanine aminotransferase (ALP), T-bilirubin, direct bilirubin, albumin, international normalized ratio (INR), prothrombin time (PT), partial thromboplastin time (PTT), creatinine, blood urea nitrogen (BUN) at the time of admission, and duration of hospital stay were collected retrospectively from patient records then stored on spreadsheet for further analysis. The data is collected and summarized in ***Table 1*** and included comorbidities and duration of hospital stay. For the final analysis, laboratory tests with missing data were excluded. The study was conducted according to the World Medical Association Declaration of Helsinki and was approved by the Medical Research Committee at HMC (MRC-01-20-823).

**Table 1:** Data collected from patient files.


PATIENTS BASELINE CHARACTERISTICS	MALE	FEMALE	P-VALUE

**Gender**	213/294	81/294	NA

**Age (years) (mean ± SD)**	49.57 ± 16.1	56.12 ± 20.18	0.004

**Weight (Kg) (mean ± SD)**	1.69 ± 0.07	1.57 ± 0.06	0.001

**Height (cm) (mean ± SD)**	73.65 ± 14.72	69.28 ± 16.58	0.057

**BMI (mean ± SD)**	25.87 ± 4.45	28.26 ± 6.67	0.002

**Neutrophil-to-Lymphocyte Ratio (mean ± SD)**	15.13 ± 14.52	18.14 ± 19.43	0.151

**Platelet-to-lymphocyte Ratio (mean ± SD)**	320.39 ± 263.08	384.20 ± 390.24	0.108

**White blood cell counts (×10^3^/ml) (mean ± SD)**	12.33 ± 5.28	13.10 ± 7.46	0.319

**Hemoglobin (g/dL) (mean ± SD)**	13.15 ± 2.23	11.61 ± 2.18	0.001

**Neutrophils (×10^3^/ml) (mean ± SD)**	10.22 ± 5.13	11.09 ± 6.98	0.247

**Lymphocytes (×10^3^/ml) (mean ± SD)**	1.13 ± .811	1.07 ± 0.79	0.543

**Lactic acid (mmol/L) (mean ± SD)**	2.28 ± 1.15	2.36 ± 1.89	0.763

**Alanine aminotransferase (U/L) (mean ± SD)**	182.94 ± 154.13	246.36 ± 285.87	0.015

**Aspartate aminotransferase (U/L) (mean ± SD)**	152.78 ± 136.14	267.99 ± 378.16	0.001

**Alkaline Phosphatase (U/L) (mean ± SD)**	305.06 ± 264.66	312.59 ± 202.70	0.817

**Total bilirubin (µmol/L) (mean ± SD)**	89.93 ± 68.62	77.08 ± 78.60	0.170

**Direct bilirubin (µmol/L) (mean ± SD)**	66.36 ± 48.70	71.77 ± 70.90	0.525

**Albumin (g/L) (mean ± SD)**	33.32 ± 7.64	31.97 ± 8.16	0.186

**International normalized ratio (mean ± SD)**	3.14 ± 23.063	1.50 ± 1.88	0.560

**Prothrombin time (mean ± SD)**	14.41 ± 19.56	13.17 ± 7.58	0.610

**Partial thromboplastin time (mean ± SD)**	33.78 ± 38.32	28.81 ± 6.99	0.292

**Creatinine (mmol/L) (mean ± SD)**	96.25 ± 64.89	99.71 ± 96.30	0.729

**Blood urea nitrogen (mmol/L) (mean ± SD)**	5.57 ± 4.11	6.21 ± 5.24	0.277

**Length of Hospital Stay (days) (mean ± SD)**	10.72 ± 9.78	11.16 ± 10.47	0.740


NA: Not applicable.

### Statistical analysis

A multivariate and univariate analysis of laboratory parameters in accordance to mortality were performed. *IBM SPSS 23®* were used to analyze the continuous variables using T-test, ANOVA test, and COX-regression for survival analysis to obtain the odds ratio (OR) with 95% confidence Interval (CI). The cut-off threshold of T-bilirubin was determined by calculating the area under the receiver operating characteristic (ROC) Curve. P-value under <0.05 was considered significant value.

## Result

**Figure 1:**
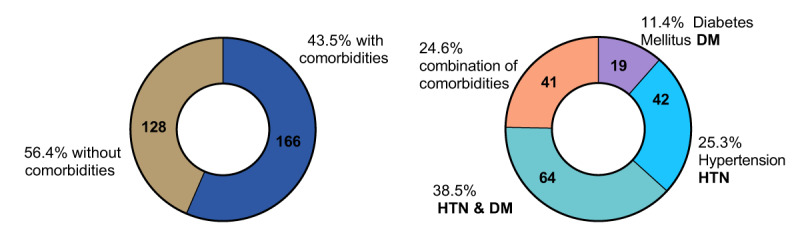
Comorbidities for AC patients enrolled in this study.

There were 213 male and 81 female patients and mean age ± SD of patients was 49.57 ± 16.1 (M = 48) and 56.12 ± 20.18 (M = 57) respectively. 31.9% patients were found older than 60 years of age and 35% patients were found between 30-45 years of age. The baseline characteristics of patients including the laboratory results of this study were collected in ***Table 1***. 166 patients (56.4%) had comorbidities. 42 patients (25.3%) had hypertension (HTN), 19 (11.4%) had diabetes mellitus (DM), 64 (38.5%) had combination of HTN and DM, 41 (24.6%) had combination of comorbidities as coronary artery disease (CAD), dyslipidemia, cholangiocarcinoma, and chronic kidney disease (CKD). 128 (43.5%) did not have a comorbid disease as shown in ***Figure 1***. The overall mortality rate was found 3.8% (n = 11).

The cumulative survival and non-survival of this study for the entire cohort is shown in ***Table 2***. Mean survival and mortality age were 50.97 ± 17.42 and 61.54 ± 18.28 respectively. There was a 96.25% (n = 283) chance of surviving with a mean hospital stay 10.04 ± 8.39 days, whereas only 3.74% (n = 11) and 31.45 ± 20.94 days for non-survivals.

**Table 2:** Demographic data and baseline parameters between survival and non-survival groups.


PARAMETERS	SURVIVAL (N = 283)	NON-SURVIVAL (N = 11)	P VALUE

**Age**	50.97 ± 17.42	61.54 ± 18.28	0.05

**Gender**			

Male (n/%)	72.8% (n = 206)	63.6% (n = 7)	0.5

Female (n/%)	27.2% (n = 77)	36.4% (n = 4)	

**PLR** (mean ± SD)	340.69 ± 308.217	267.73 ± 157.98	0.436

**NLR** (mean ± SD)	16.062 ± 16.109	13.462 ± 157.98	0.599

**Total Bilirubin (µmol/L)** (mean ± SD)	83.09 ± 67.24	170.69 ± 120.74	0.037

**Hospital stay (days)** (mean ± SD)	10.04 ± 8.39	31.45 ± 20.949	0.007


Data as mean ± SD or number (%). SD = standard deviation. NLR = Neutrophil-to-Lymphocyte Ratio. PLR = Platelet-to-lymphocyte Ratio.

**Figure 2:**
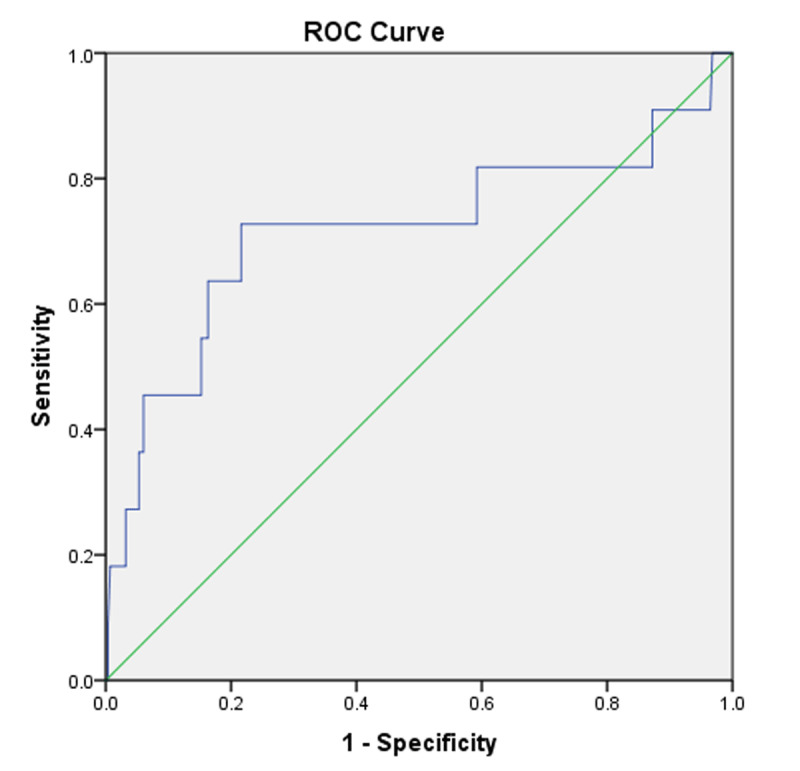
ROC analysis of T-bilirubin in AC patients in the retrospective cohort.

In multivariate logistic regression analysis using enter method, T-bilirubin found to be a significant predictor of mortality (p < 0.001), while NLR, PLR exhibited no significance (***Table 3***). In COX-regression survival analysis, T-bilirubin, age, PLR and NLR were identified in ***Figure 2***. T-bilirubin level and age were found statistically significant (p < 0.05) to predict mortality giving Hazardous Ratio (HR) 1.010, respectively. The area under ROC curve for T-bilirubin level was 0.717 (95% CI, 6.25–168.9) and this is consistent with the Cut-off point for more than or equal to 38.6 µmol/L (~2.26 mg/dL) (***Table 3***).

**Table 3:** Independent marker predictors of mortality in AC identified by multivariate COX-regression survival analysis.


MARKERS	P VALUE (2-TAILED)	STANDARD ERROR	HAZARDOUS RATIO	(95% CI)

**T-bilirubin** (Cut-off ≥ 38.6 µmol/L)	0.037	0.003	1.010	1.004–1.017

**Age**	0.004	0.030	1.090	1.029–1.155

**PLR**	0.436	0.004	0.995	0.988–1.002

**NLR**	0.599	0.032	0.968	0.910–1.030


## Discussion

The delay in diagnosis and treatment of patients with AC will conceivably aggravate the infection rate and favor the sepsis development, which results in organ failure and shortening patients’ life. Number of studies have been reported to determine factors predicting mortality in AC. Although inflammatory biomarkers have been widely used as lymphocyte count, NLR, PLR, serum albumin, creatinine, and CRP, it still have false positive, and negatives results in diagnosis. For instance, Leese et. al. reported a study on the management of acute cholangitis, 94 patients were admitted with AC and enrolled in the study. 60 were women and 34 were men with mean age of 69.7 years and for 20 days hospital stay. 15 deaths (16%) were reported within 30 days of admission. They found significantly low levels in serum albumin, and high levels in creatinine and urea in AC mortality group compared to the survivors group [17]. Liver abscess, renal failure, liver cirrhosis, malignant stricture, percutaneous transhepatic cholangiography, age older than 50 years and female sex were also reported as prognostic factors for mortality in AC [18]. Procalcitonin (PCT), WBC and CRP have been widely used as well and performed similar discriminative analysis power in prediction the severity of AC [19,20]. In 2018, Beliaev et. al. published a retrospective cohort study of 34 patients with AC and found that lymphocyte count, C-reactive protein (CRP) and neutrophil-to-lymphocyte ratio (NLR) had the highest discriminative analysis powers in diagnosing AC [20].

Same year, Tez and coworkers reported a study of 104 patients with acute suppurative cholangitis (ASC) and proposed a simple scoring system identifying mortality risk in ASC using basic clinical and laboratory tests. They identified red cell distribution width (RDW) as a factor in univariate analysis to predict mortality in cholangitis. Other variables were also identified statistically significant (p < 0.05) in multivariant analysis as well such as T-bilirubin level, and intensive care unit admission [21,22]. In 2016, Schneider et. al. published a retrospective study to create a risk prediction model for AC. 810 patients were selected, and 981 cholangitis episodes were analyzed. 22 predictors, including T-bilirubin, were achieved with good sensitivity and specificity, giving the best validated model performance to predict mortality in AC [23]. Moreover, Lin et. al. found in their retrospective cohort study a clear correlation between neutrophil to lymphocytes ratio and 1 year mortality rate in primary biliary cholangitis [24]. In 2020, Daojun and co-workers reported a study aimed to explore biomarkers for AC with or without sepsis. 65 patients with AC were enrolled in the study and 76 inpatients without AC were selected as control group. PCT was found the best to distinguish sepsis with AC and CRP was found the best to suggest an infection. Soluble triggering receptor expressed on myeloid cells 1 (sTREM-1) was found to be the best biomarker to monitor the response of AC patients to antimicrobial therapy and biliary drainage [25].

More recently, Beliaev and coworkers reported a retrospective cohort study on 212 patients after liver transplantation (LT). 30 LT patients were found with AC and 30 LT patients without AC were randomly selected as a control group. CRP, WBC, lymphocyte and NLR were found to have the highest discriminative powers for diagnosis of AC. CRP was reported to have the best discriminative analysis power among LT patients for diagnosis AC and the optimal cut-off point was equal or above 9.5 mg/L [26]. In 2021, Lavillegrand et. al. reported a retrospective multicenter study included 382 adult patients in intensive care units diagnosed with AC from 2005 to 2018. They identified different risk factors for mortality using multivariate analysis and found T-bilirubin level as one of the predictive factors of mortality [27].

Our study also identified T-bilirubin as a factor predicting mortality in AC. It is not surprising as well that duration of hospital stay would contribute to mortality giving a statistically significant result. There was no statistically significant relationship between PLR and NLR ratio with AC mortality in our study. Hyperbilirubinemia is used as a diagnostic factor in AC, also it is an indicator of cholangitis severity according to TG13/18 especially when its level >5 mg/dl (>85.5 μmol/L) [6]. Here in our study and also in others, the T-bilirubin is found to be a predictor of mortality as: First, the T-bilirubin is an indicator of severity. Second, the bile duct obstruction could be more sever and lasted for longer duration to raise the bilirubin to higher levels. Furthermore, it indicates the consequence injury to the liver caused by the biliary tree infection and impaired bilirubin excretion process. Finally, to consider the toxic effects of bilirubin itself on deferent organs of the body. These observations could be the justification of correlation between higher T-bilirubin levels and high mortality rate in AC. Consequently, we could say that T-bilirubin is a marker and could be used to predict severity in AC, however more upcoming future studies should be conducted on T-bilirubin as a marker for AC severity. Although good clinical outcomes were observed from this study, the limitations should be considered. Our results represented a retrospective cross-sectional design in which we have no control on the sample size (power of analysis) and from a single-center study.

## Conclusion

Herein, we suggest a T-bilirubin serum level as a new biomarker to identify risk of mortality in AC using basic laboratory test that is available in most primary care facilities. T-bilirubin levels is found to be largely related to short-term in AC. Clinically, high T-bilirubin level may potentiate a concern regarding poor prognosis and increase mortality risk. Further studies are still needed with larger cohorts to shed more light in these findings.
